# Critical questions in development of targeted nanoparticle therapeutics

**DOI:** 10.1093/rb/rbw011

**Published:** 2016-03-21

**Authors:** Richard Korsmeyer

**Affiliations:** Pharmaceutical Sciences, Pfizer Worldwide R&D, 445 Eastern Point Rd, Groton, CT 06340, USA

**Keywords:** nanoparticles, targeting, biodistribution, manufacturing

## Abstract

One of the fourteen Grand Challenges for Engineering articulated by the US National Academy of Engineering is ‘Engineer Better Medicines’. Although there are many ways that better medicines could be engineered, one of the most promising ideas is to improve our ability to deliver the therapeutic molecule more precisely to the desired target. Most conventional drug delivery methods (oral absorption, intravenous infusion etc.) result in systemic exposure to the therapeutic molecule, which places severe constraints on the types of molecules that can be used. A molecule administered by systemic delivery must be effective at low concentrations in the target tissue, yet safe everywhere else in the body. If drug carriers could be developed to deliver therapeutic molecules selectively to the desired target, it should be possible to greatly improve safety and efficacy of therapy. Nanoparticles (and related nanostructures, such as liposomes, nanoemulsions, micelles and dendrimers) are an attractive drug carrier concept because they can be made from a variety of materials engineered to have properties that allow loading and precise delivery of bound therapeutic molecules. The field of targeted nanoparticles has been extraordinarily active in the academic realm, with thousands of articles published over the last few years. Many of these publications seem to demonstrate very promising results in *in vitro* studies and even in animal models. In addition, a handful of human clinical trials are in progress. Yet, the biopharmaceutical industry has been relatively slow to make major investments in targeted nanoparticle development programs, despite a clear desire to introduce innovative new therapies to the market. What is the reason for such caution? Some degree of caution is no doubt due to the use of novel materials and the unproven nature of targeted nanoparticle technology, but many other unproven technologies have generated intense interest at various times. We believe that the major barrier to the exploration of nanoparticles is because they are so complex. The very design flexibility that makes the nanoparticle approach attractive also makes it challenging. Fortunately, continuing progress in experimental tools has greatly improved the ability to study biology and potential interventions at a nanoscale. Thus, it is increasingly possible to answer detailed questions about how nanoparticles can and should work. However, a detailed understanding at the mechanistic level is only the beginning. Any new medicine must not only work at the molecular level, but must also be manufactured reproducibly at scale and proven in the clinic. New materials will require new methods at all scales. The purpose of this short article is to focus on a set of questions that are being asked in the large biopharmaceutical companies and that must be answered if targeted nanoparticles are to become the medicines of the 21st century.

## Grand challenge

One of the oldest challenges in medicine is how to make a drug that does exactly what it is intended to do and only that, with no off-target action or side-effects. Indeed, this is practically the definition of an ideal drug. In practice, all drugs have limitations to their efficacy and can almost always be associated with some kind of undesired effects if the dose is sufficiently escalated. Modern drugs are invented and developed with a particular detailed molecular mechanism of action (MOA) in mind. Any given MOA has its own set positive and negative attributes which ultimately constrain the therapeutic profile associated with manipulating it, and real drugs can often have more than one MOA. (Here the term MOA refers both to the desired mechanism and to any undesired or unknown mechanisms.) The challenge for the drug designer is to achieve potency and selectivity at the desired target with a molecule that possesses all the other attributes necessary to become a practical drug [1–4]. This is a complex, multivariable optimization exercise in biology and chemistry that can strain human creativity and organizational resources, and often fails to achieve its therapeutic and commercial aim of creating an important new treatment to alleviate human suffering. It would be a great advance if it were possible to direct the therapeutic molecule selectively to the desired target, avoiding all other potential sites of interaction.

## Is a ‘magic bullet’ possible?

The concept of targeted delivery is an old one and is best known as the ‘magic bullet’, a term used by Paul Ehrlich ca. 1907, (5) inspired by the story, ‘*Der Freischutz*’, in which a hunter possesses magic bullets that hit only the intended target. In contrast to the hunter of the story, drug designers do not have the ability to point and shoot at their target, and must rely on the physical–chemical properties of the drug to interact with the complex biologic milieu and hope for a desirable effect. Any scientist working in the area knows that optimism is not always justified. For example, a drug that relies on its inherent lipophilicity to partition into a cell membrane and reach its target will encounter many other lipophilic sites as it circulates in the body. Conventional therapy relies on dosing sufficient concentrations to ensure that the desired target collects enough of the therapeutic agent. This ‘excess’ dose is often responsible for adverse events. Although it is inspiring to imagine a drug delivery system that hits only a selected target, we should also ask:

## Is there a magic target?

This question is probably as crucial as any and requires answering the more general question of what even constitutes a target. It is also necessary to differentiate between the specific cellular/molecular mechanism, i.e. the point of therapeutic intervention and other targets that might be used only for the purpose of achieving selectivity of binding. Many of the cell-surface proteins used for targeting are present in many kinds of cells, so the challenge is also to hit a subset of the available targets. Most successful efforts to date have to been to identify targets that are over-expressed in some subset of cells (e.g. tumors) and rely on binding constants for selectivity.

## Why are nano-carriers the magic bullet?

Nanoscale (i.e. < 900 nm; desirably 10–100 nm) carriers, including solid nanoparticles, nanoemulsions, liposomes, micelles, dendrimers and other such structures are the right size to interact with cells and offer the potential of great versatility in designing different targeting concepts. For the purposes of this discussion, we envision the Magic Bullet to include the following components:

A scaffold or substrate of some kind. This can be a polymeric particle, inorganic particle, liposome, micelle, dendrimer, macromolecule etc. to which are added

A targeting ligand such as a peptide, protein, or other moiety that can bind to a desired target. This targeting ligand may be covalently attached (in which case a linker is needed) or bound by intermolecular forces according to the nature of the substrate.

The active agent. A molecule or functional group of any type, that is designed to interact with a cellular or molecular mechanism, which may be different from the target used by the targeting ligand. As with the targeting ligand, the active agent may be incorporated into the nano-carrier by various mechanisms which may or may not involve additional components such as linker chemistry.

Optionally, there may also be a need for other functional components, most notably ‘stealth’ technology such as PEGylation, whose purpose is to optimize *in* *vivo* performance (e.g. clearance rate) of the formulated nano-carrier.

This four-component design concept (and there are many others) has been sufficient to provide a rich field for academic investigation and creativity, resulting in hundreds of thousands of publications on many aspects of nano-carrier design, characterization and performance, encompassing research in biology, chemistry, materials science, pharmacology, metabolism, toxicology, medicine and related fields. Despite this worldwide effort, there are only a few commercial products that would even be considered in a discussion of targeted nano-carriers and essentially none that meet all the criteria for a Magic Bullet (see sidebar). This prompts many to ask [6]:

## Why are there so few products?

Despite the number of publications over the last decade, the science and technology of nano-carriers is still developing. However great progress has been made and continues to be made. In particular, tools for imaging nano-carriers *in* *vivo* have enabled much better understanding of what can be achieved [7]. These advances are promising with respect to the prospect of rational design and (eventual) rapid development of nano-carrier-based therapeutics, but much more progress is still needed.

## Can targeting solve the problem of overall biodistribution?

Although many elegant targeting concepts have been demonstrated in the laboratory, there is still the problem of overall biodistribution of drug [8] A systemically administered therapeutic agent—targeted or not—faces variety of barriers and clearance mechanisms that all impede the delivery of agent to the intended target. Very small particles (<10 nm) are rapidly cleared by the kidneys [9]. Large particles are likely to be swept up in the Mononuclear Phagocytic System (MPS) system via opsonization and macrophage uptake (see also below). The clearance via the MPS can be mitigated by ‘stealth’ technologies such as PEGylation, Although there are limitations to the amount of PEG that can be safely administered, PEGylations remains a very viable strategy [10]. Even with good nanoparticle design, much of the mass of administered particles is liable to end up in liver and spleen [11, 12]. The degree to which targeting strategies can shift the overall mass distribution of the administered dose is still an unanswered question. At present, it is not generally possible to direct the bulk of the administered dose to the desired target tissue while ensuring that only a minimal fraction goes to non-target areas.

## Complexity

One of the most significant considerations in development of any product is the proposed complexity of the design and manufacturing process. Even the simplest nano-carriers are complex products by pharmaceutical industry standards. The fully functional four-component vision articulated above would be much more complex than any product with which the major pharmaceutical companies have experience. The author believes that complexity is the biggest barrier to adoption by industry. It should be noted that it is largely the very versatility of the nano-carrier approach, combined with the desire to attain fine selectivity that leads to such complexity. In the academic realm, it would seem from the literature, as well as from personal conversations, that much research is focused on increasingly complex designs as new discoveries are made and the associated issues are addressed. This trend appears to be driven by the natural tendency of both academic researchers and (importantly) their funding sources to emphasize ever increasingly sophisticated work over older, simpler approaches. Although sophistication and complexity are both expected and necessary, especially in academic research, they are barriers to adoption by industry. From the standpoint of industrialization, the simpler, the better. Thus, a critical question is:

## How complex must a targeted delivery system be? or how can we make it simpler?

This is not an easy question to answer. On one level, a ‘simple’ design is one with the fewest parts. Most traditional pharmaceutical formulations are a mixture of drugs and excipients that are chosen from tradition and precedent and mixed according to established art. The product is monolithic (or nearly so), and every component affects the performance of the product, which is also dependent on the processing. An alternate approach is to engineer the product with components that perform their respective functions with only the required interactions between them. By way of illustration, the traditional formulation approach is analogous to baking a cake while the engineering approach is analogous to building a bicycle. A cake may appear simpler than a bicycle, but it is much easier to confidently design alterations and improvements to the components of the bicycle than to the components of the cake. Most workers in the field of targeted nanoparticles are taking the engineering approach, but most of the pharmaceutical industry still thinks in terms of ‘formulation’.

Reformulating ‘old’ drugs with nanotechnology can improve safety

Doxil

- Reformulation of doxorubicin (liposomes)
- Decreased cardiotoxicity compared with free drug
- Marketed drug

Abraxane

- Reformulation of paclitaxel (albumin bound)
- Decreased immunotoxicity compared with free drug
- Marketed drug

Aurimune

- Reformulation of TNFa (Au nanoparticles)
- Immunotoxicity decreased by 3-fold
- In phase II clinical trials.

One promising simplification being pursued by many workers is targeting by size alone. Many solid tumors and inflamed tissues have loose endothelial junctions, leading to Enhanced Permeability and Retention of circulating nanoparticles—generally called the ‘EPR Effect’ [13]. The EPR mechanism is passive and does not require targeting ligands attached to the nanoparticles. However, this approach is controversial.

## How useful is the EPR effect?

The EPR effect has been demonstrated many times in animal tumor (xenograft) models and is believed to be common in human tumors as well. However, it also appears to vary across different tumor types and critics have questioned whether it is sufficiently universal to form a basis of a targeted therapy. Importantly, the same particle characteristics that facilitate the EPR effect for tumors also lead to accumulation of particles in the liver and spleen, so it is unlikely that EPR alone can achieve full selectivity.

## What size is best?

It depends on the application. In general, smaller size facilitates tissue penetration, but smaller particles tend to be rapidly cleared. Larger particles can pack more functionality (in principle), but then are more subject to clearance by MPS unless ‘stealthed’. A recent model published by Wittrup *et al.* [14] suggests that the optimal size for particles intended to accumulate in tumors is around 20 nm—similar to an IgG.

## What is the best material?

Nanoparticles have been made of lipids, polymers, DNA, carbon, metals, metal oxides and other materials. There has been considerable work done with materials that have precedent in other applications, such as poly(lactic-co-glycolic acid). At this time, it is not possible to say, with certainty what constitutes the ‘best’ material. However, it may be worth considering that the most ‘precedented’ materials owe this status to their history in applications other than nanoparticles, and therefore may not be the optimum materials to use.

## How should drug be incorporated?

As with antibody-drug conjugates, many nanoparticle approaches utilize covalent attachment of the drug to the scaffold. This provides control over stability and release mechanism, but requires additional investments in both the chemistry of the scaffold and of any needed linking groups. Introducing a covalent bond can add regulatory complexity because covalent attachment of a known molecule to another creates a New Chemical Entity, with all the regulatory considerations that implies.

Phase Partitioning is a common loading mechanism that relies on thermodynamically favorable interactions between the drug and carrier, as with loading a lipophilic drug into a lipid emulsion. This is the method most familiar to the pharmaceutical industry, but it can be quite limiting. The nature of partitioning means that there will usually be an unbound fraction that leads to an initial burst of release. Delivery systems that depend on phase partitioning for drug loading almost always exhibit sustained-release (i.e. non-selective) of the drug in addition to release due to any incorporated targeting mechanism.

## Should we have safety concerns about engineered nano-structured carriers?

Although a nanoparticle approach can reduce toxicity of a drug, in other cases significant problems have been observed with engineered nanomaterials [15–17]. Some of these problems represent vulnerabilities common to all injectable products and others are related to the particulate nature of the materials.

As with all sterile injectable products, it is necessary to ensure not only sterility but the absence of endotoxin or any pyrogenic components. Perhaps because nanoparticles often utilize non-standard materials and are more complex than traditional products, this has been an area where problems have commonly been flagged. Thoughtful quality specifications for raw materials and other ingoing components (including process water) and maintenance of sterility and/or terminal sterilization during processing are areas meriting detailed attention.

Unlike traditional injectable medicines, nanoparticles are the right size to interact with cells and are structured to do so. As with other foreign materials, nanoparticles can be expected to rapidly bind to serum proteins, depending on the surface properties of the particle. Certain proteins mark the particle for uptake by phagocytic cells, a process called opsonization [8]. When the opsonized cells are taken up by the MPS, they can be expected to accumulate in the liver, spleen, and lymph nodes. Those organs are therefore primary areas of scrutiny for any adverse effects, in addition to any other areas that might be associated with the specific mechanism under test.

Nano-carriers are often PEGylated to reduce MPS uptake, but this tactic has limitations. Complement activation has been observed with PEGylated liposomes and can be a dose-limiting toxicity [18–20].

Opsonins are not the only serum proteins that can interact with nanoparticles. The components of the coagulation cascade can also bind to the particle surface and trigger thrombogenesis [21]. In extreme cases, the result can be disseminated intravascular coagulation, a syndrome characterized by clotting mechanisms being activated throughout the body [22].

Hemolysis screening is part of routine toxicology testing with nanoparticles and has been frequently observed, especially with cationic, surfactant and metal oxide particles [17].

In summary, although nanoparticles have been associated with several problematic observations, there has been no unique toxicity associated with nanoparticles *per se*.

## Can these sophisticated systems be manufactured?

The answer to this question has to be yes, but perhaps not by familiar methods. Process design must be planned early in development. Because the field is relatively new, the overwhelming majority of published results with targeted systems have been with systems prepared at laboratory scale. Only a few commercial companies have advanced to the point of manufacturing nano-carrier-based products, and of those even fewer incorporate targeting. There are at least three approaches that could be taken to scale-up:

- adapt some form of a ‘laboratory’ method to commercial batch process equipment or customized batch process equipment. This is the most common method in the pharmaceutical industry.
- Develop a small-scale continuous process and scale up by increasing run times (for modest scale up) and by scaling the continuous equipment (for even larger scales). This method is growing in popularity in the pharmaceutical industry because of the advantages it offers in capital costs and flexible capacity.
- A third approach that has not yet been highly developed would be to develop a small-scale continuous process that can be replicated on a massively parallel basis, thereby ‘eliminating’ scale-up. It would be a tremendous advantage if it were possible to do ‘screening’ experiments at small scale but using processes that can either be scaled up, or which do not require scale-up. Microfluidics techniques may offer some opportunities for very small, but finely controlled processes that can be replicated very cheaply on a massive scale thereby ensuring that products produced at various scales are in fact equivalent.

In conclusion, targeted nano-carriers offer the promise of the Magic Bullet therapy that has been dreamed of for over a century, but from an industrial view, many questions remain. Continuing work by a number of groups is beginning to answer these critical questions, but acceptance of this mode of therapy will come only after success has been repeatedly demonstrated.
